# Cloning of Three Aflatoxin B1 Oxidases of the Dipeptidyl Peptidase III Family and Evaluation of Their Potential for Practical Applications as Decontamination Enzymes

**DOI:** 10.3390/toxins16100419

**Published:** 2024-09-27

**Authors:** Igor Sinelnikov, Ivan Zorov, Yury Denisenko, Kristina Demidova, Alexandra Rozhkova, Larisa Shcherbakova

**Affiliations:** 1 Federal Research Centre “Fundamentals of Biotechnology” of the Russian Academy of Sciences (RAS), 119071 Moscow, Russia; inzorov@mail.ru (I.Z.); denisenkoyura@mail.ru (Y.D.); krisdemid@bk.ru (K.D.); a.rojkova@fbras.ru (A.R.); 2 All-Russian Research Institute of Phytopathology of RAS, Bolshie Vyazemy, 143050 Moscow, Russia

**Keywords:** aflatoxin B1, recombinant enzymes, aflatoxin oxidases, dipeptidyl peptidase III, enzymatic toxin degradation, *Pleurotus eryngii*, *Lentinula edodes*, *Cantharellus cibarius*, *Armillaria tabescens*

## Abstract

Aflatoxin B1 (AFB1) produced by some *Aspergillus* species belongs to the most dangerous contaminants of animal feeds. Development of safe and cost efficient decontamination methods saving feed quality and nutritional value are of paramount importance. The use of recombinant AFB1-detoxifying microbial enzymes represents a promising biotechnological approach meeting the aforementioned requirements. In this study, three AFB1-degrading oxidases (AFOs) from edible basidiomycetes *Cantharellus cibarius*, *Lentinula edodes* and *Pleurotus eryngii* as well as AFO from *Armillaria tabescens* were expressed in *E. coli* Rosetta (DE3) and purified by immobilized metal-chelate chromatography. The stabilizing effect of the addition of glycerol and β-mercaptoethanol during protein extraction is shown. The catalytic constants of the recombinant AFOs (rAFOs) and other characteristics, which might be important for their practical application (and optimal temperature and pH, thermolability, regulation of the activity by metal ions and chelating agents, storage stability) were investigated. Among the obtained enzymes, rAFO from *P. eryngii* (Pe-AFO), which was characterized by the highest specific activity, thermostability and pH stability (especially at acidic pH range), the lowest K_m_, and relative resistance to the inhibition by phytate, showed the best AFB1-degrading efficacy. However, Pe-AFO and all other rAFOs significantly decreased the target activity during heating above 45 °C, storage frozen or lyophilization.

## 1. Introduction

Mycotoxins of different chemical nature are produced by many fungi as secondary metabolites, and aflatoxins are among the most dangerous of them [1]. Aflatoxins of B- and G-types are synthesized by several *Aspergillus* species, mainly by widespread *Aspergillus flavus* and *A. parasiticus*, which are known to be the most frequently encountered contaminants of feed and food products worldwide [2]. The majority of toxigenic *A. flavus* strains produce aflatoxin B1 (AFB1), a highly toxic compound that impairs feed and food safety and poses a serious threat to human and animal health [3].

Suppression of AFB1 producers’ development during feed storage by maintenance of correct conditions, in particular, avoiding excess moisture, along with strict temperature control can prevent aflatoxin contamination, but these conditions are not always properly maintained that may result in insufficient effective suppression of toxigenic fungal strains. Moreover, forage grain and feed raw materials can be polluted with AFB1 before drying and being placed into storage, e.g., already during harvesting due to contact with toxigenic *Aspergilli*. Because of the rigorous world restrictions of the AFB1 residues in feeds (5 µg/kg in the European Union [4] or 20 µg/kg in the US and Customs Union countries [Technical Regulations of the (CU) 015/2011]), even a small excess over the maximum permissible concentrations of the toxin content in feeds can cause their condemnation and result in significant economic losses. Therefore, decontamination of plant raw stuff and feeds, including forage grain, is a matter of primary importance.

To detoxify AFB1, various physical, chemical, or biological methods are used [5]. Although this toxin is difficult to degrade in contaminated products due to its high thermal stability, some physical methods such as strong heating or pressure can lead to its destruction, demonstrating sufficient efficiency. For instance, a treatment by autoclaving can reduce AFB1 content in contaminated rice by 75% [6]. However, most effective physical methods are quite expensive, require a significant amount of energy, and elaborate equipment [7]. Chemical detoxification is often carried out under very aggressive conditions, involving reagents that significantly worsen the quality and nutrient value of the exposed products, sometimes making them unsuitable for their intended use [8].

Along with physical and chemical methods, biotechnological approaches for AFB1 degradation attract considerable attention due to their advantages, such as the possibility of products becoming decontaminated at temperatures acceptable for product processing, minimal losses in the product quality, environmental friendliness, and scalability. Biotechnologies for reducing AFB1 content in contaminated products can be divided into three main categories: (1) application of consortia or individual strains of destructor microorganisms, (2) the use of plant or fungal extracts [9], and (3) treatments with toxin-degrading/detoxifying enzymes, including recombinant ones. For example, extracts of certain herbs (*Hybanthus enneaspermus*, *Eclipta prostrata*, and *Centella asiatica*) were reported to degrade over 70% of AFB1 within 48 h of incubation [10]. Cultivation of *Pleurotus ostreatus* on substrates contaminated with AFB1 and other aflatoxins resulted in a significant reduction of their contamination [11]. Fungal extracts containing intracellular toxin-detoxifying enzymes are well documented as possible tools for feed and food decontamination [9,12]. For instance, extracts from the basidiomycete *Pleurotus eryngii* were able to degrade more than 50% of AFB1 after 1 day of incubation at 25 °C [9].

Since aflatoxin contamination of foodstuffs represents a global challenge and no universal solution for detoxification is currently available despite various chemical, physical, and biological methods developed, it is imperative to continue studies in this direction to provide effective and environmentally friendly decontamination of agricultural products [13]. At present, application of recombinant enzymes appears to be one of the most promising methods for the removal of various mycotoxins, including AFB1, from agricultural products. These technologies have been demonstrated to be effective in cleaving AFB1. A number of examples exist of laccases [14] and other oxidases [15] that have demonstrated efficacy in neutralizing aflatoxins. In 1998 [16], a novel enzyme named aflatoxin-oxidase (AFO) with high affinity for the bisfuran ring was successfully isolated from *Armillaria tabescens*, followed by successful expression in the yeast Pichia pastoris in 2011 [17,18,19]. The AFO enzyme was found to be capable of catalyzing the opening and subsequent hydrolysis of the bisfuran ring. Tomin et al. conducted a comprehensive computational investigation to examine the affinity of an AFO from *A. tabescens* for AFB1 and elucidate the structural details of the enzyme [20]. The results of this study provide compelling evidence that AFO belongs to the dipeptidyl peptidase III (DPP III) family, which, in addition to AFB1-tranforming activity, also has peptidase activity. It was subsequently demonstrated that the catalytic center of DPP III is capable of binding to and degrading AFB1. Despite some studies disputing the possibility of AFB1 binding to the catalytic center [21] and declaring the absence of aflatoxin-degrading activity [22], it can be stated with confidence that this family of enzymes, particularly DPP III from basidiomycetes, is effective at degrading AFB1, as demonstrated in laboratory conditions using both model systems and real substrates [23,24].

Nevertheless, despite the described potential of DPP III as an enzyme to reduce mycotoxin contamination of agricultural products, the technological properties of this enzyme make it poorly suited for practical use [13]. The DPP III family of enzymes is intracellularly localized and lacks high stability under extracellular conditions [25]. The pioneering works that describe the thermal-stability properties of AFO from *A. tabescens* emphasize that the enzyme’s half-life at 30 °C is 90 min. In other studies, various members of DPP III also demonstrate extremely low stability [26,27]. Currently, there are no reliable data on the influence of structure on stability, which makes it impossible to model stable forms of this enzyme. DPP III from basidiomycetes is traditionally considered the most effective enzyme for degrading AFB1 and is referred to in the literature as aflatoxin oxidase (AFO). However, information on other representatives of the AFO-type enzymes is scarce, despite the presence of their homologues in numerous basidiomycetes. A homologue of aflatoxin oxidase from *Trametes versicolor* [28], with properties similar to those of the enzyme from *A. tabescens*, has been described. To fully comprehend the degradation mechanisms and enhance the target activity and stability of such enzymes, it is necessary to search for novel members of this family and analyze their biochemical properties and activity under controlled and reproducible experiments.

In the present study, three new AFB1-degrading oxidases (AFOs) from *Pleurotus eryngii* (Pe_AFO), *Lentinula edodes* (Le_AFO), and *Cantharellus cibarius* (Cc_AFO) were first cloned and as well as aflatoxin oxidase from *A. tabescens* (At_AFO), were expressed in *E. coli* Rosetta (DE3). To assess the potential of these recombinant AFOs for practical application, their catalytic constants and other characteristics, such as optimal temperature and pH, thermolability, resistance to chelating agents, regulation of the target activity by metal ions, and storage stability were investigated.

## 2. Results

### 2.1. Cloning and Isolation of AFO-Encoding Genes

Three genes encoding of full-length ORFs of *pe-afo* (2022 b.p.), *le-afo* (2181 b.p.) and *cc-afo* (2217 b.p.) were amplified using primer pairs, designed based on the whole genome sequences of *P. eryngii* (GenBank GCA_029467805.1), *L. edodes* (GCA_021015755.1), *C. cibarius* (GCA_003521295.1), respectively, which are available in the NCBI database. For amplification of AFO from *A. tabescens* (*at-afo*), a synthetic gene (GeneBank AAX53114.1) was used as a matrix [23]. Then, *pe-afo*, *le-afo*, *cc-afo* and *at-afo* PCR products of studied cDNAs (Figure S1, see Supplementary Materials) were cloned into the pET-28a vector under the control of the T7 promoter using ligation independent cloning (LIC), and then inserted into *E. coli Rosetta* (DE3). Expression plasmids containing the coding sequences of the corresponding target genes were obtained and denoted as pET-peAfo, pET-leAfo, pET-ccAfo and pET-atAfo, respectively (Figure S2).

### 2.2. In Silico Analysis of rAFO

**Figure 1:**
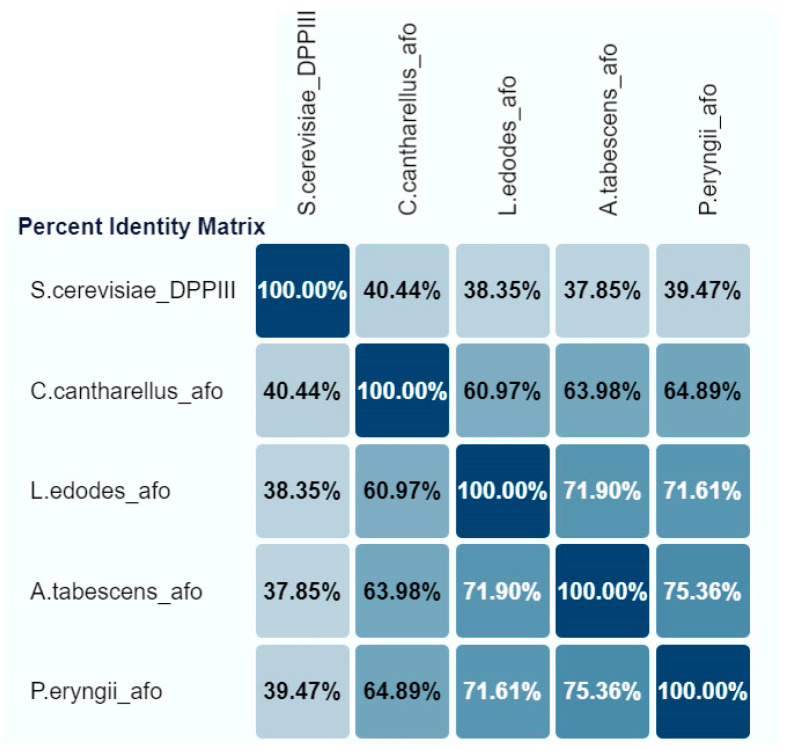
The percent identify matrix of recombinant AFOs expressed in *E. coli* Rosetta (DE3).

The three cloned genes (*pe-afo*, *le-afo*, *cc-afo*) were compared with the “reference” aflatoxin oxidase gene from *A. tabescens* (*at-afo*). All rAFOs investigated were monomeric proteins with an identify ranging from about 61 to 75% (Figure 1).

Thus, the BLASTn analysis of the recombinant Cc-AFO, Le-AFO and Pe-AFO revealed a 63.98%, 71.90% and 75.36% identity with At-AFO, respectively (Figure 1). In contrast to Pe-AFO and At-AFO, Le-AFO and Cc-AFO were observed to have an additional site of 34 and 37 amino acids at the N-terminal ends. The absence of a detectable signal peptide and the intracellular location of these proteins indicate that the function of this N-terminal site remains unclear. In addition, a high variability was observed in the N-terminal regions of all obtained enzymes, while the centers of the protein globules were highly conservative. The catalytic domains and their surrounding regions exhibited 100% identity, with the catalytically relevant “EECRA” motif and the two histidines in the “HEXXGH” motif presented in all amino acid sequences (Figure S3, see Supplementary Materials). The homology with the reference DPPIII from *Saccharomyces cerevisiae* (PDB: 3CSK) was low and did not exceed 40%. All conserved regions corresponding to M49 family proteins (DPPIII) were observed in the cloned sequences.

### 2.3. rAFO Expression

**Figure 2:**
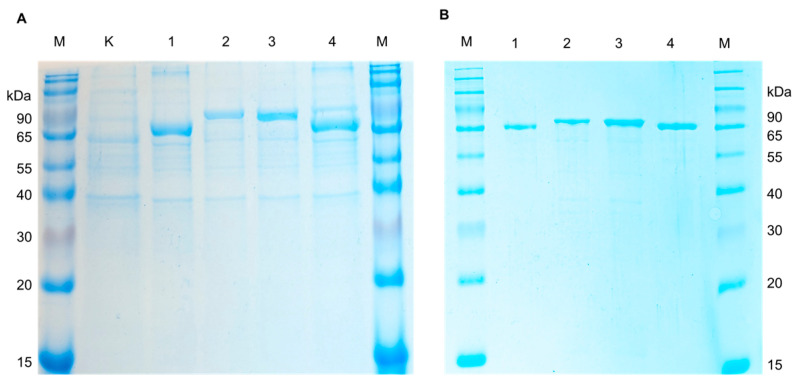
The SDS-PAGE electropherogram of the (**A**) crude lysates of *E. coli* Rosetta (DE3) cells (K—non-induced control; 1, Pe-AFO; 2, Cc_AFO; 3, Le_AFO; 4, At-AFO; and M—molecular weight marker) and (**B**) recombinant AFOs purified from *E. coli* Rosetta (DE3) cells (1, Pe-AFO; 2, Cc_AFO; 3, Le_AFO; 4, At-AFO; and M—molecular weight marker).

For expression of recombinant AFOs in *E. coli*, the strain Rosetta (DE3) carrying additional tRNA necessary for the expression of eukaryotic proteins in procaryotes was used. The transformants harboring pET-peAfo, pET-leAfo, pET-ccAfo and pET-atAfo were cultivated in Terrific Broth (supplemented 50 µg/mL of kanamycin). After induction with isopropyl-β-D-thiogalactopyranoside (IPTG), the cultures were disrupted by ultrasonication, and the recombinant AFOs (rAFOs) were purified from the soluble fraction of bacterial cell lysates by immobilized metal-chelate chromatography (IMAC). Isolation and purification of the enzymes to homogeneity were achieved using a one-step chromatographic procedure similar to all rAFOs as described in the Materials and Methods section (see the Section 4.2). Purified rAFOs were analyzed by SDS PAAG (12.5% acrylamide/bisacrylamide) under reducing conditions. Figure 2 shows a Coomassie-stained SDS polyacrylamide gel with the purified rAFOs, which had a molecular mass close to 78 kDa.

### 2.4. Determination of Biochemical Parameters and Stability of Recombinant AFOs

The primary objective of the study was to identify and characterize new members of the DPPIII enzyme family from basidiomycetes. To achieve this, a series of experiments were conducted, including the determination of catalytic activity, Km, temperature and pH optimum, temperature and pH stability, the influence of ions, chelating agents (ethylenediaminetetraacetic acid (EDTA) and sodium phytate), and tests on the effect of additives on the storage of the enzyme solutions.

**Figure 3:**
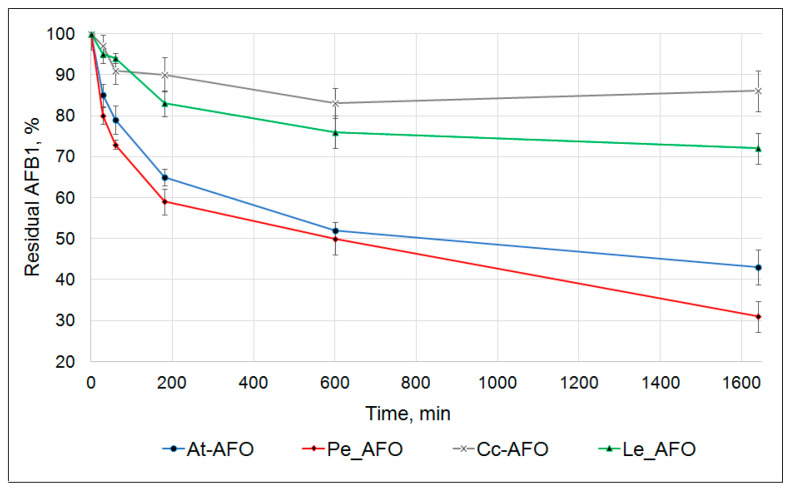
Kinetics of AFB1 degradation by four recombinant AFOs.

Firstly, the total catalytic activity towards AFB1 was determined under conditions commonly indicated as optimal for AFO from *A. tabescens* and other known fungal AFOs [17,28]. The rAFO from *P. eryngii* (Pe-AFO) showed a higher activity of 298 Units/mg under the aforementioned conditions, compared to the activity (185 units/mg) observed for At-AFO (Table 1). Conversely, the specific AFB1-degrading activities of rAFOs from *L. edodes* and *C. cibarius* were significantly lower than the specific activity of the oxidase from *A. tabescens*. However, our further experiments on the degradation dynamics (Figure 3) demonstrated that the observed low levels of the specific activity could be in more degree apparent than real due to an insufficient stability of the tested enzymes in spite of them being incubated with AFB1under optimal conditions.

In these experiments, the toxin degradation curves were determined at pH 6.0 and temperature of 30 °C within 24 h. The starting AFB1 concentration and the dosage of each recombinant enzyme amounted to 0.5 µg/mL and 1 mg/mL, respectively. The main decrease in AFB1 concentration occurred in the first 3 h of the reaction. Subsequently, the rate of the toxin degradation progressively slowed down. The result of this was that none of the rAFOs were capable of exhaustive AFB1 removal from the reaction mixture. Nevertheless, rAFOs from *P. eryngii* and *A. tabescens* were able to degrade, respectively, about 70% and 60% of AFB1 within 24 h. The rAFOs from *L. edodes* and *C. cibarius* were characterized by a much less effective degradation pattern with only 30% reduction in AFB1 concentration during the same period (Figure 3).

**Figure 4:**
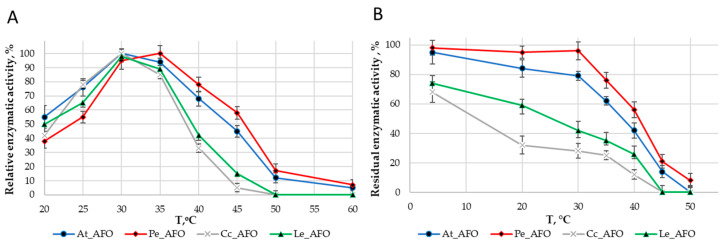
Effect of temperature on the activity (**A**) and stability (**B**) rAFOs.

**Figure 5:**
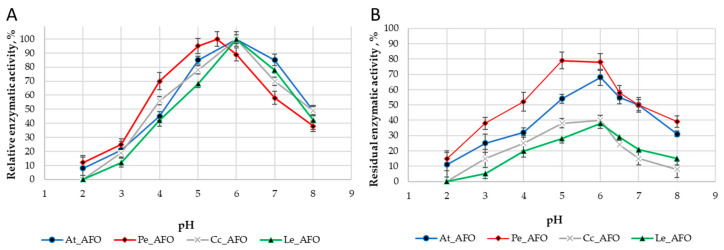
pH optimum (**A**) and pH stability (**B**) of four rAFOs.

For each enzyme, the optimal temperature (Figure 4A) and optimal pH (Figure 5A) were determined. The optimum activity for all enzymes was in the range of 30–35 °C. The optimal activity was determined at pH 6.0 for all AFOs except Pe_AFO, for which the optimum was determined to be 5.5.

The thermostability of the enzymes was evaluated by residual activity following 30 min of incubation at temperatures ranging from 4 to 50 °C (Figure 4B). Pe-AFO and At-AFO had similar temperature inactivation profiles and exhibited some toxin-degrading activity at 45 °C, while the less effective Cc-AFO and Le-AFO were rapidly inactivated already once the temperature rose above 30 °C. Moreover, the two last enzymes lost more than 30% of their target activity even after 30 min incubation at 4 °C. Such rapid inactivation of rAFOs from *L. edodes* and *C. cibarius* could be the reason for their failed real activity towards AFB1.

Thus, rAFOs from *A. tabescens* and *P. eryngii* isolated under these conditions exhibited the highest temperature stability, retaining 50% of activity after incubation at 40–45 °C with an optimum at 35 °C, while the optimal temperature for rAFOs from *L. edodes* and *C. cibarius* remained at the level of 30 °C.

The DPIII family, to which the enzymes under study belong, is traditionally characterized by a narrow optimum of action in the pH range of 5.0–7.5 [25], that is associated with their intracellular localization. Accordingly, obtained rAFOs exhibited a similar pH-dependent profile of inactivation (Figure 5).

**Figure 6:**
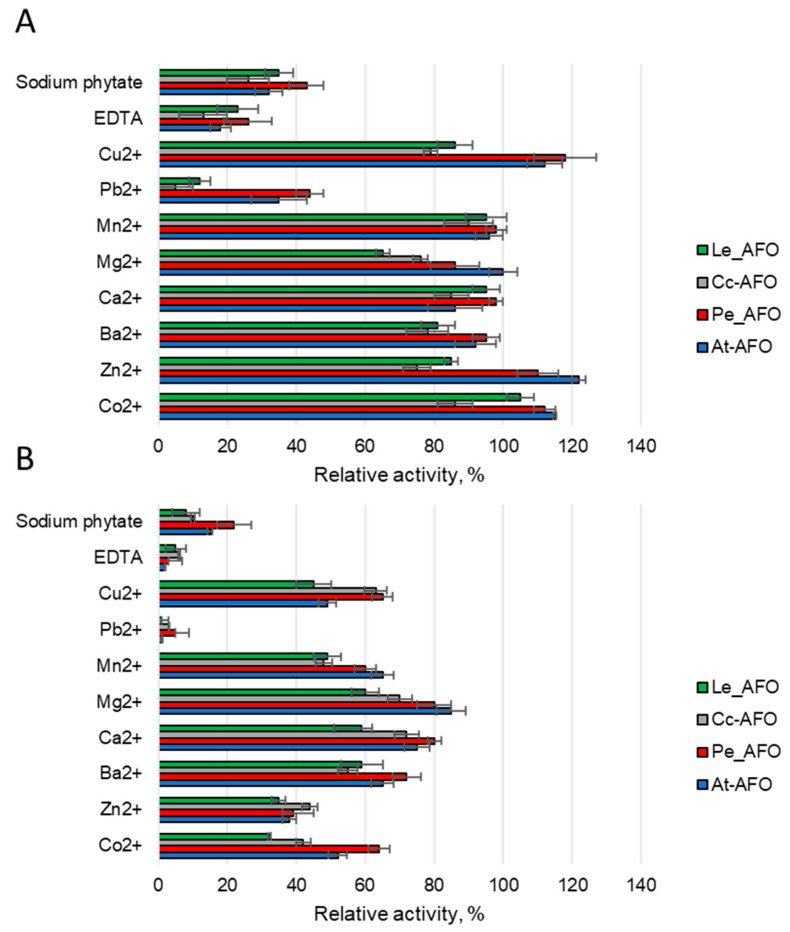
The effect of various metal ions and two chelator agents at concentrations of 1 (**A**) and 5 mM (**B**) on AFB1 degrading activity induced by AFOs.

To evaluate the effect of metals on the AFB1-degrading activity of rAFOs, different ions or two chelating compounds at concentrations of 1 or 5 mM were added to the reaction media (Figure 6). Predictably, it was found that rAFOs as metalloenzymes [25] were sensitive to metal chelators. Thus, the catalytic activity, which reduced by 60–80% upon addition of 1 mM of EDTA or completely inhibited by 5 mM of this agent, was completely restored when an equimolar concentration of zinc, manganese or cobalt ions was added. This suggests that manganese or cobalt can replace zinc in the catalytic center of the enzymes. The addition of sodium phytate had an effect similar to the effect of 1 mM EDTA and reduced the AFB1-degrading activity by an average of 60% (Figure 6). Among the rAFOs, Pe-AFO, which demonstrated the highest target activity, was the most weakly inhibited by sodium phytate. Such relative resistance to this chelator might give the enzyme an advantage when treating grain or other raw materials with a high phytate content. Mg^2+^, Ba^2+^ and Ca^2+^ ions rather reduced or had no effect on the target activity. Increasing the concentration of Co^2+^, Zn^2+^ and Mn^2+^ above 1 mM has an inhibitory effect, and reduces the toxin-degrading activity of all rAFOs by 30–40%.

**Figure 7:**
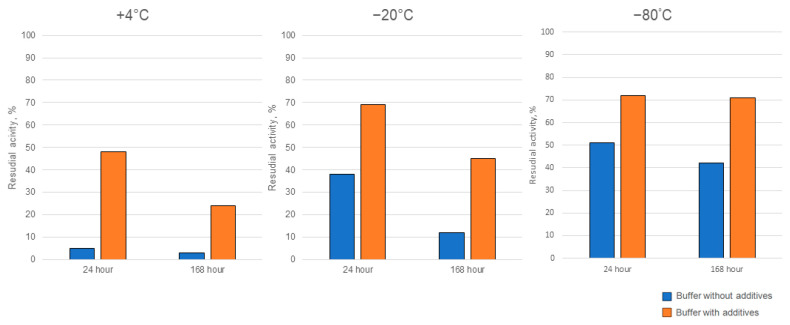
The improved storage stability of rAFO from *C. cibarius* (Cc-AFO) by the addition of two additives (glycerol and β-mercaptoethanol) to the isolation and storage buffers.

Ensuring the enzymes’ activity is stable during storage is a crucial aspect that determines feasibility for practical applications outside the laboratory setting. Therefore, we carried out a series of storage experiments, in which Cc-AFO as the enzyme with the greatest thermally induced instability was used. We tested the target activity of Cc-AFO samples, which were stored at +4 °C or frozen (at −80 °C or −20 °C) in buffers without additives or with glycerol and β-mercaptoethanol, which were supposed to stabilize the enzyme. In addition, we examined AFB1-dergading activity of the freeze-dried Cc-AFO samples. The addition of glycerol and β-mercaptoethanol to the samples stored at +4 °C for 7 days improved the enzyme stability, resulting in a 10-fold increase in the degrading activity compared to the samples stored without these additives under the same conditions. The glycerol and β-mercaptoethanol added to the samples prior to their freezing at −20 and −80 °C also increased the Cc-AFO stability that resulted in a two-fold improvement in the AFB1-degrading activity after one-week-long storage (Figure 7). During the lyophilization process, the enzyme could have lost up to 70%, but it remained stable during subsequent storage at −20 °C until the end of experiments. Similar properties were observed in Pe_AFO. In this experiment, 90% of the Pe_AFO activity still remained in the case of using a storage buffer containing glycerin and β-mercaptoethanol and a temperature of 4 °C for 24 h. The storage of the enzyme at −20 °C without these additives as well as its freeze drying resulted in a reduction of its AFB1-degrading activity by 25 and 32%, respectively.

## 3. Discussion

To our knowledge, at least three enzymes referred to as oxidases isolated from xylotrophic basidiomycetes (*Cerrena unicolor*, *A. tabescens* and *Trametes versicolor)* have been reported to have the ability of AFB1 degradation [21,29,30]. Moreover, AFOs from *T. versicolor* and *A. tabescens* expressed in yeast were shown to be able to detoxify peanuts [30], and cereal grain contaminated with AFB1 [23]. One of the first studies of AFO from *A. tabescens* demonstrates low temperature stability [17]. However, this enzyme remains the gold standard in the study of AFB1 degradation.

In the present study, three AFOs that represented homologous proteins from three species of edible mushrooms (*C. cibarius*, *L. edodes* and *P. eryngii*) as well as AFO from *A. tabescens,* which was used as a reference, were successfully expressed in *E. coli*, and their AFB1-degrading potential was studied under different conditions.

All the studied enzymes with oxidase activity simultaneously belonged to peptidases of the DPPIII family and have catalytic centers with similar structures [31]. A lot of knowledge has been accumulated about the representatives of this DPPIII family. In particular, their broad and mobile catalytic center has been demonstrated to successfully perform AFB1 degradation [20,21]. Despite the high degree of structural variation observed within the DPIII family, they all possess a conserved active center and similar biochemical characteristics, with optimal pHs and temperatures around 5.5–7.0 and 30–40 °C, respectively [25]. In general, enzymes produced by basidiomycetes are characterized by more acidic pH than DPPIII from mammals or yeast, which show the highest activity at alkaline pH values between 7.5 and 8.5 [32,33]. The observed homogeneity of biochemical characteristics can be attributed to the similar conditions that prevail within eukaryotic cells and the analogous functions inside the cell.

Despite similarities in the area surrounding the catalytic center (see Figure 1), the AFB1-degrading activity of the obtained recombinant enzymes was different. AFOs from *L. edodes* and *C. cibarius* had low activity, while the activity of AFO from *P. eryngii* was comparable to the AFO from *A. tabescens* or exceeded that. Earlier studies showed that the lower K_m_ value of AFOs is favorable for AFB1 degradation [34]. In our study, Pe-AFO was characterized by the lowest K_m_. The revealed difference in K_m_ values between Pe-AFO and At-AFO indicated a higher substrate specificity of Pe-AFO, which, however, had a weak effect on the completeness of AFB1 degradation in 24 h tests.

It should be noted that the relatively quick loss of activity at temperatures above 40 °C and low stability during storage [35,36] are probably properties of many DPPIII enzymes. The temperature-dependent changes in the AFB1-degradation dynamics of rAFOs in the range of 4–50 °C revealed in our experiments coincided with corresponding changes characteristic for other members of the DPPIII family, but the rate of inactivation of rAFOs from *L. edodes* and *C. cibarius* significantly exceeds the degradation rates shown for AFOs from other organisms including basidiomycetes [25,32].

Supplementation of isolation and storage buffers with the additives stabilized the enzymes and slowed down their degradation, but it did not completely stop it. Thus, isolation of rAFOs in the presence of glycerol and mercaptoethanol may be a way to decrease the enzymes’ inactivation by temperatures used during feedstuff processing. It should be noted that the protectants contained in the final eluate are only in trace amounts, which can be easily removed in full, e.g., by a quick dialysis. The protective effect of β-mercaptoethanol and glycerol are in good agreement with previously published data for DPPIII [37], which may indicate a common mechanism of degradation of this class of enzymes. DPPIII are conserved intracellular proteins [38] with a similar domain structure and role in the cell, which most likely causes poor stability in solutions. Given that the approaches to stabilization work equally well for mammalian and basidiomycete DPPIII, it can be assumed that they are universal for all representatives of the class.

Ions of Zn^2+^, Mn^2+^, Cu^2+^ and Co^2+^ at low concentrations have a weak activating effect, whereas when their concentration increases, the enzyme inhibition occurs [25]. Also, like all metal-dependent enzymes, the investigated rAFOs were inhibited by EDTA, which chelates metal ions [37]. A similar effect resulted from sodium phytate, which significantly reduced the activity of rAFOs, probably due to the binding of zinc ions of the active center. The relative resistance of the most active rAFOs, Pe-AFO, to sodium phytate action might give the enzyme an advantage when treating grain or other raw materials with high phytate content. In general, our results towards the ions effect on AFOs’ activity are in good agreement with earlier studies, but the data on the positive effect of barium ions were not confirmed [18].

Our experiments, as well as other studies, specifically aimed at investigating the completeness of AFB1 degradation showed that AFOs do not provide an exhaustive destruction of the mycotoxin [20,25,31]. This is presumably due to the rapid inactivation of enzymes rather than for the achievement of kinetic equilibrium or poor substrate availability. For instance, an incomplete AFB1 removal from a model solution and an insufficient decontamination of grain substrates strongly polluted with the toxin were observed when using a recombinant AFO from *A. tabescens* in spite of the treatment time prolongation [23].

At the same time, AFB1 content reduction below the limits that are permissible in a number of countries [4] was achieved after treatments of medium-contaminated wheat and corn grain with a high concentration of this enzyme under controlled conditions [23]. In addition, rAFOs from *T. versicolor* expressed in *S. cerevisiae* or *P. pastoris* were shown to effectively and quickly reduce AFB1 and AFB2 contamination of rice grain or peanut, respectively [28,30]. All these findings and results of the current study on one of the novel rAFOs, Pe-AFO, confirm suggestions that AFB1-degrading oxidases can represent potential decontamination agents. Since enzymatic treatments including feed mixing with various enzymatic additives are used during preparation of complete fodders for animals, AFB1-degrading enzymes might be also added at this stage of fodder treatment. Therefore, enzymatic decontamination could be included into the technological process to provide additional benefits for production of AFB1-free feed products safe for animals. However, the technological characteristics of the obtained enzymes so far do not allow us to confidently consider them as ready-to-use components of decontamination preparations. The low temperature stability, loss of the target activity during storage and freeze drying, and the sensitivity to phytate usually presented in cereal grain create significant obstacles to the practical application of these rAFOs. On the other hand, the high variability of the area surrounding their catalytic center and the structural diversity of DPPIIIs allows good opportunities for engineering the improved enzymes with desired technological properties. However, there are currently no data on amino acid substitutions leading to the stabilization of the DPPIII protein globule. It is likely that the search and testing of DPPIII homologues from prokaryotic cells may assist in the development of new, more stable enzyme variants. A real chance of this is already exemplified by a recent isolation of DPPIII from a thermophilic microorganism, which retains activity at 50 °C [39]. Collectively, this fact, the availability of the aforementioned rAFOs [23,28,30], and also a useful balance between similarities and variabilities of AFOs from basidiomycetes warrant the expediency of further investigations required to stabilize AFOs and improve their AFB1-degrading efficacy.

## 4. Materials and Methods

### 4.1. Cloning AFOs and Building of Expression Plasmids

The coordinates of the AFOs homologue in the *Pleurotus eryngii* (GenBank GCA_029467805.1), *Lentinula edodes* (*GCA_021015755.1*), and *Cantharellus cibarius* (GCA_003521295.1) genomes were determined using the BLAST algorithm [40]. The reading frame was predicted using AUGUSTUS [41]. Service sequences were added to the 5′ ends of specific oligonucleotide primers for Lic-cloning in modification vector pET28a.

*P. eryngii* (VKM-F-2402), *L. edodes* (VKM F-1999), and *C. cibarius* (wild strain selected in the Moscow region) were cultured on Czapek-Dox Modified Broth for 7 days. Total RNA was isolated from 100 mg of fresh mycelium, using the RNeasy Plant Mini Kit (Qiagen, Redwood City, CA, USA), as recommended by the manufacturer.

The first cDNA strand was synthesized using the reverse transcription Maxima H Minus Reverse Transcriptase (Thermo Fisher Scientific, Waltham, MA, USA) with a dT18 primer. Specific cDNA amplification was carried out by PCR using the oligonucleotide primers (Table 2) and 2 μL of the first-strand cDNA. A synthetic gene described in previous work was used as a matrix to clone *afo* from *A. tabescens* [42].

The reaction mixture contained polymerase buffer, 0.2 mM of each primer, 1.5 mM MgCl_2_, 0.2 mM of each deoxynucleotide, and 2 units of Phanta Max Super-Fidelity DNA Polymerase (Vazyme, China). The PCR reactions to amplify *pe-afo*, *le-afo*, *cc-afo* and *at-afo* were carried out using the following program: 95 °C for 3 min; 32 cycles of 95 °C for 15 s; 55 °C for 30 s and 72 °C for 2 min; and a final extension of 72 °C for 5 min. The PCR products were cloned into the pET28 vector by LIC-cloning [43] with C-terminal hexahistidine tag.

### 4.2. Enzyme Expression and Purification

Tryptone, yeast extract, glycerol, kanamycin sulphate and IPTG were purchased from HiMedia Laboratories (Thane, India).

AFO constructs were transformed into *E. coli* Rosetta and cultured in Terrific broth (TB) medium to OD_600_ equals to 0.5 at 37 °C. Expression of recombinant proteins was induced by 0.2 mM IPTG and cells were further grown at 18 °C for 24 h. Cells were separated by centrifugation at 5500 g for 10 min and resuspended in lysis buffer (20 mM K-PO_4_ buffer, pH 7.4, 500 mM NaCl, 20 mM imidazole, 1 mM β-mercaptoethanol and 10% glycerol). The suspension underwent sonication, followed by centrifugation at 10,000× *g* for 30 min at 4 °C.

The recombinant AFOs (rAFO) were purified from the culture supernatants by IMAC to obtain homogeneous rAFO samples. The culture fluids were applied to a 15 mL Ni-Sepharose Excel column (Cytiva, Marlborough, MA, USA) at a flow rate of 1.5 mL/min following centrifugation (10 min, 21,300 rpm). The column was then equilibrated with an equilibration buffer (20 mM K-PO_4_ buffer, pH 7.4, 250 mM NaCl, 1 mM β-mercaptoethanol and 10% glycerol). To remove non-specifically bound proteins, the column was washed with wash buffer (20 mM K-PO_4_ buffer, pH 7.4, 500 mM NaCl, 40 mM imidazole, 1 mM β-mercaptoethanol and 10% glycerol) at a flow rate of 2.5 mL/min. The rAFOs were eluted in a single step with potassium phosphate buffer (20 mM K-PO_4_ buffer, pH 7.4, 500 mM NaCl, 250 mM imidazole, 1 mM β-mercaptoethanol and 10% glycerol) at a flow rate of 2 mL/min. The fractions exhibiting the highest rAFO concentration were pooled and subjected to dialysis against a buffer solution (5 mM K-PO_4_ buffer, pH 6.0, 0.5 mM β-mercaptoethanol and 10% glycerol).

To isolate recombinant enzyme forms for studying the storage stability of enzymes “without additives”, the isolation was performed using the same protocol, excluding the addition of β-mercaptoethanol and glycerol in the buffer.

### 4.3. rAFO Activity Assay

The standard technique for activity measurement in this work can be described as follows: an aliquot of enzyme is diluted with buffer to a concentration of 0.1 mg/mL. Subsequently, 400 µL of enzyme solution is mixed with 100 µL of AFB1 (0.5 µg/mL) solution and incubated at 35 °C for 30 min. The reaction is then stopped by rapid heating to 100 °C for 5 min. The reaction mixture is then cooled on ice and used for analysis.

One unit of the rAFO activity was defined as the amount of enzyme that transformed 1 pmol of AFB1 equivalent per minute. Specific activities are expressed as units per milligram of protein.

Then, activities of the rAFO were measured under the optimal conditions determined above at different AFB1 concentrations ranging from 0.1 to 1 μg/mL. The V_max_ and K_m_ values were calculated using a Lineweaver–Burk plot

### 4.4. Residual AFB1 Analysis by HPLC

The toxin-degrading activity and kinetic characteristics of rAFOs from different sources (*P. eryngii*, *L. edodes*, *C. cibarius* and *A. tabescens*) were investigated by measuring the residual AFB1 in the used enzyme-toxin reaction mixtures by reversed-phase HPLC using an Agilent 1200 system with diode array detector, recording at 275 nm; Kromasil Ethernity 5-C18 column 4.6 × 250 mm with a pre-column, flow rate 800 μL/min; gradient 40% to 68% acetonitrile in 20 mM K-PO_4_ buffer, pH 3.0 in 15 min.

### 4.5. rAFO pH Stability and Thermostability

The optimal pH for rAFOs activity was determined by performing assays at a temperature of 30 °C and ranging pH from 2 to 8 for 30 min. To estimate the enzymes stabilities at different pH values, enzymes were pre-incubated at 20 °C for 30 min in buffers at various pH values (pH 2.0–8.0). Residual enzyme activity was then measured at the previously determined optimal pH and temperature for 30 min.

The optimal temperatures for rAFOs activity were determined by performing assays at pH 6.0 and temperatures ranging from 20 °C to 70 °C for 30 min. The thermal stabilities of enzymes were evaluated by measuring residual enzyme activities under standard conditions after enzymes were pre-incubated at various temperatures (4–50 °C) for 30 min.

### 4.6. Metal Ion, Chelator and Storage Effect Testing

The tests for storage stability and the effect of metals, EDTA or sodium phytate, were carried out in a manner similar to those used for pH and temperature stability. An aliquot of the enzyme was diluted to a concentration of 1 mg/mL and various auxiliary agents were added. The final concentrations of metal and chelators were 1 mM or 5 mM, and β-mercaptoethanol and glycerol concentrations amounted to 1 mM and 10%, respectively. The treated enzyme samples were stored at temperatures of −80 °C, −20 °C, and +4 °C. In addition, the stability of the lyophilized sample prepared using a SP VirTis Freeze Dryer (SP Scientific, Warminster, PA, USA) was evaluated. A freshly isolated enzyme preparation without any additions and not subjected to a storage procedure was used as a control. The residual activity was monitored over time, with readings taken at 24 and 168 h (7 days) at pH 6.0 and 30 °C.

### 4.7. Statistics

The significance of the difference between the mean values obtained in the AFB1-degradation experiments by rAFOs were considered to be significant at *p* ≤ 0.05 (STATISTICA v. 6.1 software package; StatSoft Inc., Tulsa, OK, USA). All experiments were carried out in three replications; each experiment included three analytical replications. Y-bars on Figure 3, Figure 4, Figure 5, Figure 6 and Figure 7 indicate standard deviation (SD).

## Figures and Tables

**Table 1:** Specific activity and K_m_ values of four AFOs expressed in *E. coli* Rosetta (DE3).

Enzyme Sources	Recombinant Enzymes (Mean ± SD)	
	Activity, U/mg	K_m_, mM
*A. tabescens*	185 ± 8	3.36 ± 0.21
*P. eryngii*	298 ± 11	2.32 ± 0.15
*L. edodes*	110 ± 16	6.32 ± 0.26
*C. cibarius*	54 ± 6	10.5 ± 0.31

**Table 2:** The list of oligonucleotide primers used in the research.

Name	5′ Sequence
Plre_F	TACTTCCAATCCATGACTTTCTCCACTTCCATTC
Plre _R,	TATCCACCTTTACTTCAAAGAGAACGTTCAATGAAAC
Lede_R	TATCCACCTTTACTTCACAGTCTCCGGTCGATGAAGCTC
Lede _F	TACTTCCAATCCATGTGGAGCCTAAGGGGCCTCAGAC
Caci_R,	TATCCACCTTTACTTTAAATATCTCGCTCCACAAATG
Caci _F	TACTTCCAATCCATGCGCAGCCGGTTTCCCTT
Arta_F	TACTTCCAATCCATGGCTACTACTACAACTGTT
Arta_R	TATCCACCTTTTTACTGCAATCTTCTCTCTCAATGAAG

## Data Availability

Data are contained within the article and supplementary materials.

## Supplementary Materials

The following supporting information can be downloaded at: https://www.mdpi.com/article/10.3390/toxins16100419/s1, Figure S1: Agarose gel electrophoresis of the PCR products of cDNA from *Pleurotus eryngii*, *Lentinula edodes*, *Armillaria tabescens* and *Cantharellus cibarius*; Figure S2: Expression plasmid map. Figure S3: Multiple alignment of amino acid sequences of DPP III aflatoxin oxidases from *Saccharomyces cerevisiae*, *Cantharellus cibarius*, *Lentinula edodes*, *Pleurotus eryngii*, *Armillaria tabescens* (**a**) and their phylogenetic tree (**b**). Table S1: Amino acid sequences of AFOs from *Armilaria tabescens*, *Cantharellus cibarius*, *Pleurotus eryngii* and *Lentinula edodes.*
