# Supplementary material for: Cloning of Three Aflatoxin B1 Oxidases of the Dipeptidyl Peptidase III Family and Evaluation of Their Potential for Practical Applications as Decontamination Enzymes
Source: Toxins (Basel). 2024 Sep 27;16(10):419. doi: 10.3390/toxins16100419 (PMC11511539; doi:10.3390/toxins16100419)
Supplement: Supplementary file 1 [file toxins-16-00419-s001.zip › toxins-3168859-supplementary.pdf]

Supplementary Materials: Cloning of Three Aflatoxin B1 Oxidases of the Dipeptidyl Peptidase III Family and Evaluation of Their Potential for Practical Applications as Decontamination Enzymes

Igor Sinelnikov, Ivan Zorov, Yury Denisenko, Kristina Demidova, Alexandra Rozhkova and Larisa Shcherbakova

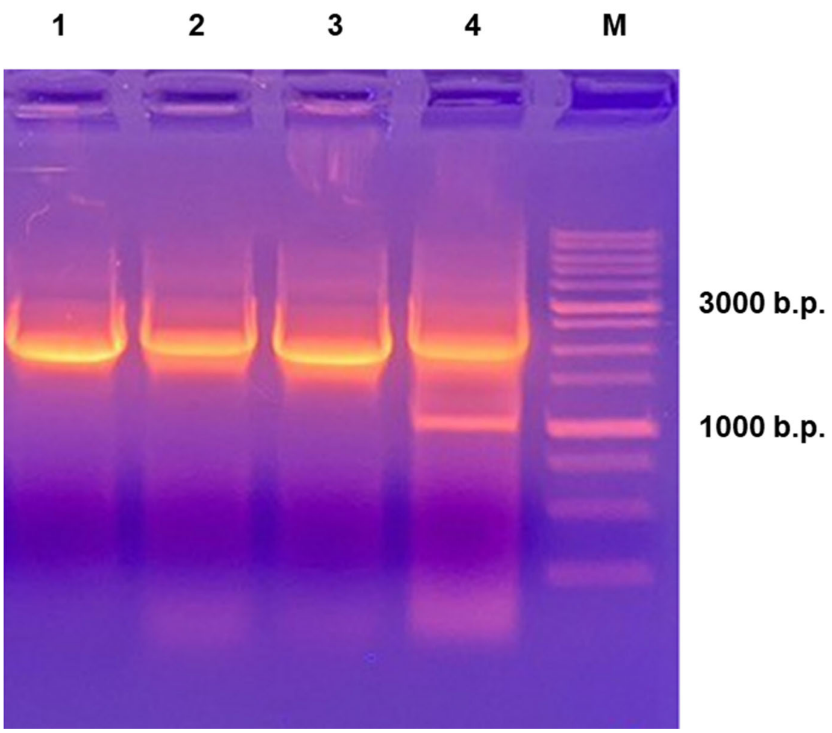

Figure S1. Agarose gel electrophoresis of the PCR products of cDNA from (1) *Pleurotus eryngii*, (2) *Lentinula edodes*, (3) *Armilaria tabescens* and (4) *Cantharellus cibarius*.

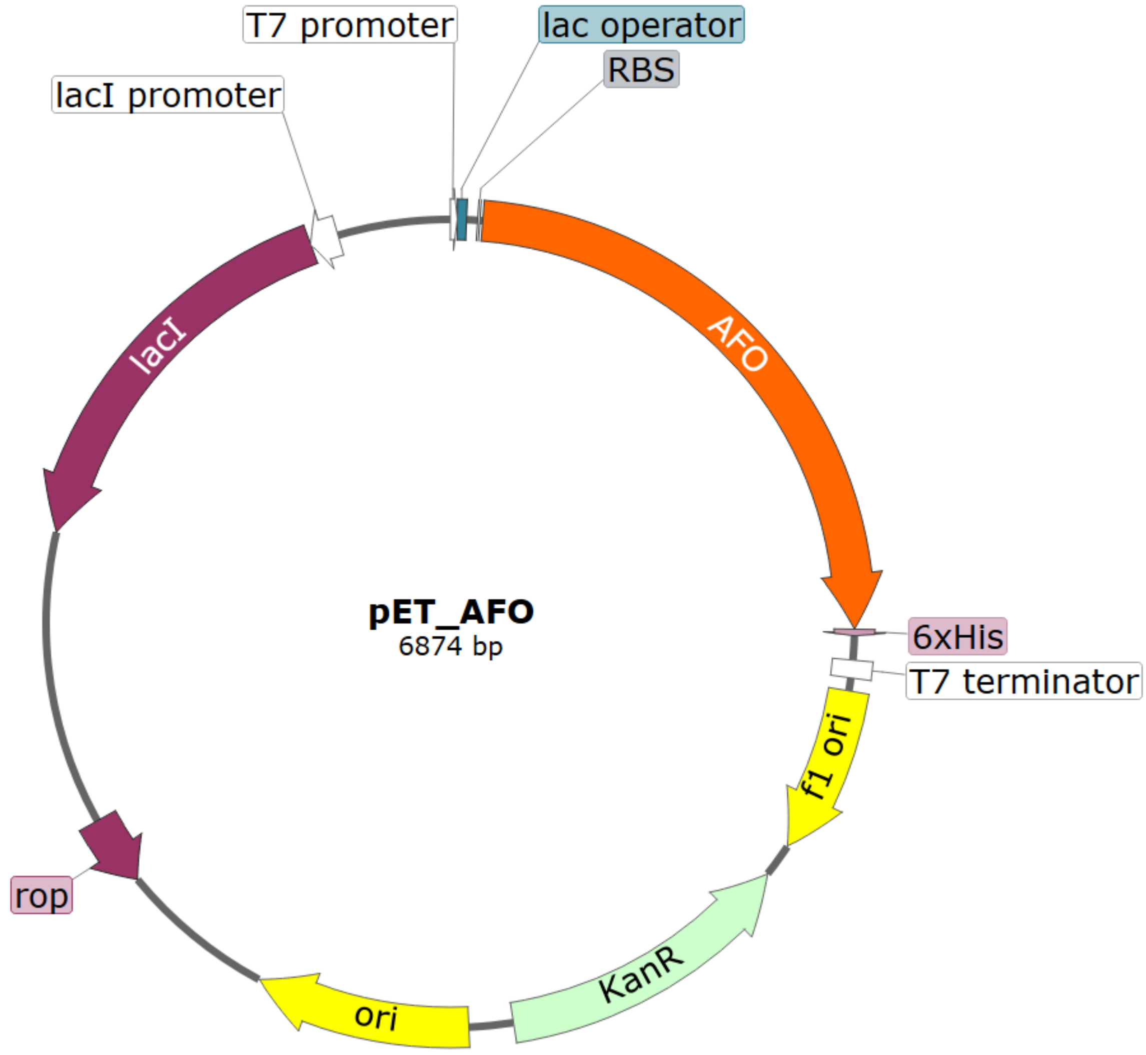

Figure S2. Expression plasmid map.

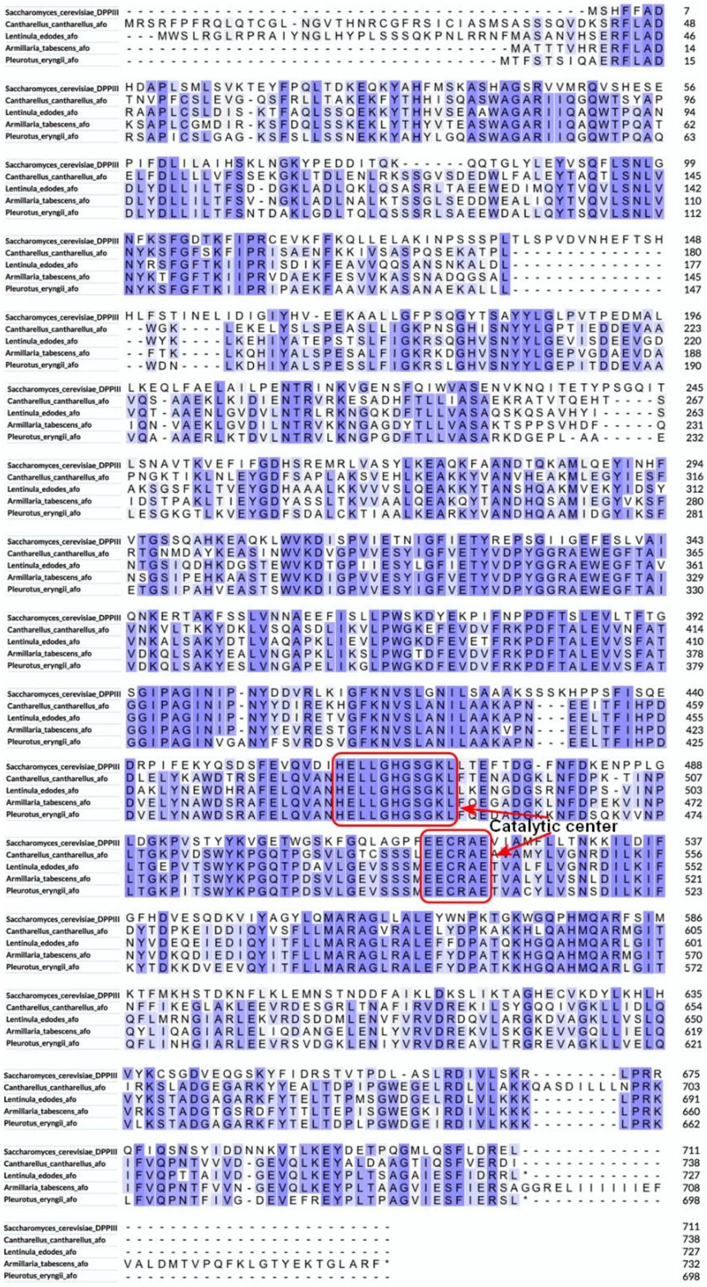

a)

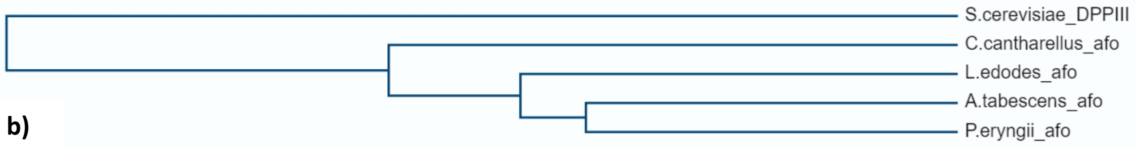

b)

Figure S3. Multiple alignment of amino acid sequences of DPP III aflatoxin oxidases from Saccharomyces cerevisiae, Cantharellus cibarius, Lentinula edodes, Pleurotus eryngii, and Armilaria tabescens (a) and their phylogenetic tree (b). Red frames indicate the catalytic center.

Table S1. Amino acid sequences of AFOs from *Armilaria tabescens*, *Cantharellus cibarius*, *Pleurotus eryngii* and *Lentinula edodes*.

Organism	Sequences
Armillaria_tabescens_afo	MATTTVHRERFLADKSAPLCGMDIRKSFDQLSSKEKLYTHYVTEASWAGARIIQAQ<br>WTPQATDLYDLLILTFSVNGKLADLNALKTSSGLSEDDWEALIQYTVQVLSNLVNY<br>KTFGFTKIIPRVDAEKFESVVKASSNADQGSALFTKLKQHIYALSPESALFIGKRKDG<br>HVSNYYLGEPVGDAEVDAIQNVAEKLGVDILNTRVKKNGAGDYTLLVASAKTSPPS<br>VHDFQIDSTPAKLTIEYGDYASSLTKVVAALQEAKQYTANDHQSAMIEGYVKSFNS<br>GSIPEHKAASTEWVKDIGPVVESYIGFVETYVDPYGGRAEWEGFTAIVDKQLSAKYE<br>ALVNGAPKLIKSLPWGTDFEVDVFRKPDFTALEVVSFATGGIPAGINIPNYYEVREST<br>GFKNVSLANILAAKVPNEELTFIHPDDVELYNAWDSRAFELQVANHELLGHGSGK<br>LFQEGADGKLNFDPEKVINPLTGKPITSWYKPGQTPDSVLGEVSSSMEECRAETVAL<br>YLVSNLDILKIFNYVDKQDIEDIQYITFLLMARAGLRALEFYDPATKKHGQAHMQA<br>RMGITQYLIQAGIARLELIQDANGELENLYVRVDREKVLSKGKEVVGQLLIELQVRK<br>STADGTGSRDFYTTLTEPISGWEGKIRDIVLKKKLPRKIFVQPNTFVVNGEVQLKEYP<br>LTAAGVIESFIERSAGGRELIIIIIIEFVALDMTVPQFKLGTYEKTGLARF
Cantharellus_cantharellus_afo	MRSRFPFRQLQTCGLNGVTHNRCGFRSICIASMSASSSQVDKSRFLADTNVPFCSLE<br>VGQSFRLLTAKEKFYTHHISQASWAGARIIQGQWTSYAPELFDLLLLVFSSEKGKLTD<br>LENLRKSSGVSDEDWLFALEYTAQTLSNLVNYKSFGFSKFIPRISAENFKKIVSASPQS<br>EKATPLWGKLEKELYSLSPEASLLIGKPNSGHISNYYLGPTIEDDEVAAVQSAAEKLK<br>IDIENTRVRKESADHFTLLIASAEKRATVTQEHTSPNGKTIKLNLEYGDFSAPLAKSV<br>EHLKEAKKYVANVHEAKMLEGYIESFRTGNMDAYKEASINWVKDVGPVVESYIGF<br>VETYVDPYGGRAEWEGFTAIVNKVLTKKYDKLVSQASDLIKVLPWGKEFEVDVFRK<br>PDFTALEVVNFATGGIPAGINIPNYYDIREKHGFKNVSLANILAAKAPNEEITFIHPD<br>DLELYKAWDTRSFELQVANHELLGHGSGKLFTENADGKLNFDPKTINPLTGKPVDS<br>WYKPGQTPGSVLGTCSSSLEECRAEAVAMYLVGNRDILKIFDYTDPKEIDDIQYVSFL<br>LMARAGVRALELYDPKAKKHLQAHMQARMGITNFFIKEGLAKLEEVRDESGRLTN<br>AFIRVDREKILSYGQQIVGKLLIDLQIRKSLADGEGARKYYEALTDPIPGWEGELRDL<br>VLAKKQASDILLLNPRKIFVQPNTVVVDGEVQLKEYALDAAGTIQSFVERDI
Pleurotus_eryngii_afo	MTFSTSIQAERFLADRSAPICSLGAGKSFSLLSSNEKKYAHYLGQASWAGARIIQGQ<br>WTPQAQDLYDLLILTFSNTDAKLGDLTQLQSSSRLSAEEWDALLQYTSQVLSNLVN<br>YKSFGFTKIIPRIPAEKFAAVVKASANAEKALLLWDNLKDHIYALSPESSLFIGKRSLG<br>HVSNYYLGEPITDDEVAAVQAAAERLKTDVLNTRVLKNGPGDFTLLVASARKDGE<br>PLAAELESGKGTLKVEYGDFSDALCKTIAALKEARKYVANDHQAAMIDGYIKSFET<br>GSIPAHVEASTSWVKDIGPVVESYIGFVETYVDPYGGRAEWEGFTAIVDKQLSAKYE<br>SLVNGAPELIKGLPWGKDFEVDVFRKPDFTALEVVSFATGGIPAGINVGANYFSVRD<br>SVGFKNVSLANILAAKAPNEELTFIHPDDVELYNAWDSRAFELQVANHELLGHGS<br>GKLFQEDADGKKNFDSQKVVNPLTGKPITSWYKPGQTPDSVLGEVSSSMEECRAET<br>VACYLVSNSDILKIFKYTDKKDVEEVQYITFLLMARAGLRALEFYDPATKKHGQAH<br>MQARLGITQFLINHGIARLEEVRSVDGKLENIYVRVDREAVLTRGREVAGKLLVELQ<br>VLKSTADGAGARKFYTELTDPLPGWDGEIRDIVLKKKLPRKLFVQPNTFIVGDEVEF<br>REYPLTPAGVIESFIERSL
Lentinula_edodes_afo	MWSLRGLRPRAIYNGLHYPLSSSQKPNLRRNFMASANVHSERFLADRAAPLCSLDIS<br>KTFAQLSSQEKKYTHHVSEAAWAGARIIQAQWTPQANDLYDLLILTFSDDGKLADL<br>QKLQSASRLTAEEWEDIMQYTVQVLSNLVNYRSFGFTKIIPRISDIKFEAVVQQSANS<br>NKALDLWYKLKEHIYATEPSTSLFIGKRSQGHVSNYYLGEVISDEEVGDVQTAAENL<br>GVDVLNTRLRKNGQKDFTLLVASAQSKQSAVHYISAKSGSFKLTVEYGDHAAALK<br>KVVVSLQEAKKYTANSHQAKMVEKYIDSYNTGSIQDHKDGSTEWVKDTGPIIESYL<br>GFIETYVDPYGGRAEWEGFTAVVNKALSAKYDTLVAQAPKLIEVLPWGKDFEVETF<br>RKPDFTALEVVSFATGGIPAGINIPNYYDIRETVGFKNVSLANILAAKAPNEELTFIHP<br>DDAKLYNEWDHRAFELQVANHELLGHGSGKLLKENGDGSRNFDPSVINPLTGEPV<br>TSWYKPGQTPDAVLGEVSSSMEECRAETVALFLVGNRDILKIFNYVDEQEIEDIQYIT<br>FLLMARAGLRALEFFDPATQKHGQAHMQARLGITQFLMRNGIARLEKVRDSDDM<br>LENVFVRVDRDQVLARGKDVAGKLLVSLQVYKSTADGAGARKFYTELTTPMSGW<br>DGELRDLVLKKKLPRKIFVQPTTAIVDGEVQLKEYPLTSAGAIESFIDRRL
